# Supplementary material for: Urban Transfer Entropy across Scales
Source: PLoS One. 2015 Jul 24;10(7):e0133780. doi: 10.1371/journal.pone.0133780 (PMC4514628; doi:10.1371/journal.pone.0133780)
Supplement: S1 File — The initial values are based on the exploration of a subset of the system’s phase space and not in a calibration procedure against any particular real urban process. The values selected here are the ones where the overall of the structures generated resembles the ones observed in the UK. The fourth column specifies if a parameter’s initial value is changed throughout the execution of the model or not. Table B.Times series for T = 4.0 showing the number of clusters generated at each scale. Table C.Times series for T = 5.0 showing the number of clusters generated at each scale. Table D.Times series for T = 6.0 showing the number of clusters generated at each scale. (DOCX) [file pone.0133780.s001.docx]

**Supplementary Information:**

**Urban transfer entropy across scales**

**Roberto Murcio, Robin Morphet, Carlos Gershenson, Michael Batty**

**S1 Table A.** List of parameters used in the urban model. The initial values are based on the exploration of a subset of the system’s phase space and not in a calibration procedure against any particular real urban process. The values selected here are the ones where the overall of the structures generated resembles the ones observed in the UK. The fourth column specifies if a parameter’s initial value is changed throughout the execution of the model or not.

| **Parameter** | **Definition** | **Initial Value** | **Change value?** |
| --- | --- | --- | --- |
| $P_{i}\left(t\right)$ | Urban potential for development in zone *i* at time *t*. | 1 or -1 selected at random | YES Eq.8 |
| *K* | Damping factor representing the influence that the local settlements has over the regional potential. | 0.1 | NO |
| $\varepsilon_{i}\left(t\right)$ | Random events representing the individual decisions take it at regional and local levels which cannot be measured directly. | 1 or -1 selected at random | YES |
| $C_{i}\left(t\right)$ | Current number of urban units at zone *i* at time *t.* | 0 | YES Eq. 11 |
| $C_{i}^{\max}\left(r\right)$ | Maximum capacity for zone *i*. | Calculated Equation 10 | NO |
| $C_{seed}^{\max}$ | Maximum capacity for seed zone | 100 | NO |
| $\kappa$ | Damping factor representing the influence that the regional settlements has over the current capacity of zone *i*. | 0.1 | NO |
| $N\left(t\right)$ | Number of migrant units at time *t.* | 0 | YES Eq. 9 |

**S1 Table B.** Times series for *T=4.0* showing the number of clusters generated at each scale

|  | **Time periods** | | | | | | | | | |
| --- | --- | --- | --- | --- | --- | --- | --- | --- | --- | --- |
| **Scale** | **t_1_** | **t_2_** | **t_3_** | **t_4_** | **t_5_** | **t_6_** | **t_7_** | **t_8_** | **t_9_** | **t_10_** |
| **1** | 87 | 234 | 399 | 568 | 737 | 907 | 1073 | 1244 | 1427 | 1618 |
| **2** | 7 | 15 | 21 | 25 | 30 | 35 | 39 | 45 | 50 | 56 |
| **3** | 6 | 11 | 14 | 16 | 17 | 18 | 19 | 20 | 21 | 23 |
| **4** | 6 | 9 | 10 | 11 | 11 | 11 | 12 | 12 | 12 | 13 |
| **5** | 5 | 7 | 8 | 8 | 8 | 8 | 8 | 9 | 9 | 9 |
| **6** | 4 | 5 | 5 | 5 | 6 | 6 | 6 | 6 | 6 | 6 |
| **7** | 3 | 4 | 4 | 5 | 5 | 5 | 5 | 5 | 5 | 5 |
| **8** | 3 | 3 | 4 | 4 | 4 | 4 | 4 | 4 | 4 | 4 |
| **9** | 3 | 3 | 3 | 3 | 3 | 3 | 3 | 3 | 3 | 3 |
| **10** | 2 | 2 | 2 | 2 | 2 | 2 | 2 | 2 | 2 | 2 |

**S1 Table C.** Times series for *T=5.0* showing the number of clusters generated at each scale

|  | **Time periods** | | | | | | | | | |
| --- | --- | --- | --- | --- | --- | --- | --- | --- | --- | --- |
| **Scale** | **t_1_** | **t_2_** | **t_3_** | **t_4_** | **t_5_** | **t_6_** | **t_7_** | **t_8_** | **t_9_** | **t_10_** |
| **1** | 51 | 87 | 154 | 230 | 315 | 408 | 502 | 607 | 732 | 873 |
| **2** | 4 | 5 | 7 | 9 | 11 | 14 | 16 | 20 | 24 | 29 |
| **3** | 3 | 4 | 6 | 7 | 8 | 9 | 10 | 11 | 12 | 13 |
| **4** | 3 | 4 | 5 | 6 | 7 | 7 | 7 | 8 | 8 | 9 |
| **5** | 3 | 4 | 5 | 5 | 4 | 6 | 6 | 6 | 7 | 7 |
| **6** | 3 | 3 | 4 | 4 | 4 | 4 | 5 | 5 | 5 | 5 |
| **7** | 3 | 3 | 3 | 4 | 4 | 4 | 4 | 4 | 4 | 4 |
| **8** | 2 | 3 | 3 | 3 | 3 | 3 | 3 | 3 | 3 | 4 |
| **9** | 2 | 3 | 3 | 3 | 3 | 3 | 3 | 3 | 3 | 3 |
| **10** | 2 | 2 | 2 | 2 | 2 | 2 | 2 | 2 | 2 | 2 |

**S1 Table D.** Times series for *T=6.0* showing the number of clusters generated at each scale

|  | **Time periods** | | | | | | | | | | |
| --- | --- | --- | --- | --- | --- | --- | --- | --- | --- | --- | --- |
| **Scale** | **t_1_** | **t_2_** | **t_3_** | **t_4_** | **t_5_** | **t_6_** | **t_7_** | **t_8_** | **t_9_** | **t_10_** |  |
| **1** | 51 | 58 | 81 | 111 | 150 | 200 | 256 | 324 | 409 | 513 |  |
| **2** | 3 | 3 | 3 | 4 | 5 | 6 | 8 | 11 | 14 | 19 |  |
| **3** | 2 | 2 | 3 | 3 | 3 | 4 | 4 | 5 | 6 | 8 |  |
| **4** | 2 | 2 | 3 | 3 | 3 | 4 | 4 | 4 | 5 | 6 |  |
| **5** | 2 | 2 | 3 | 3 | 3 | 3 | 4 | 4 | 5 | 5 |  |
| **6** | 2 | 2 | 2 | 3 | 3 | 3 | 4 | 4 | 4 | 4 |  |
| **7** | 2 | 2 | 2 | 3 | 3 | 3 | 3 | 3 | 3 | 4 |  |
| **8** | 2 | 2 | 2 | 3 | 3 | 3 | 3 | 3 | 3 | 3 |  |
| **9** | 2 | 2 | 2 | 3 | 3 | 3 | 3 | 3 | 3 | 3 |  |
| **10** | 2 | 2 | 2 | 2 | 2 | 3 | 3 | 3 | 3 | 3 |  |
